# Revisiting *Francisella tularensis* subsp. *holarctica*, Causative Agent of Tularemia in Germany With Bioinformatics: New Insights in Genome Structure, DNA Methylation and Comparative Phylogenetic Analysis

**DOI:** 10.3389/fmicb.2018.00344

**Published:** 2018-03-13

**Authors:** Anne Busch, Prasad Thomas, Eric Zuchantke, Holger Brendebach, Kerstin Neubert, Josephine Gruetzke, Sascha Al Dahouk, Martin Peters, Helmut Hotzel, Heinrich Neubauer, Herbert Tomaso

**Affiliations:** ^1^Institute of Bacterial Infections and Zoonoses, Friedrich-Loeffler-Institut, Jena, Germany; ^2^Department of Biological Safety, German Federal Institute for Risk Assessment, Berlin, Germany; ^3^Algorithmic Bioinformatics, Department of Mathematics and Computer Science, Institute of Computer Science, Freie Universität Berlin, Berlin, Germany; ^4^Standort Arnsberg, Chemisches und Veterinäruntersuchungsamt Westfalen, Arnsberg, Germany

**Keywords:** *Francisella tularensis* subsp. *holarctica*, genome analysis, tularemia, high quality genome, phylogeny

## Abstract

*Francisella* (*F.*) *tularensis* is a highly virulent, Gram-negative bacterial pathogen and the causative agent of the zoonotic disease tularemia. Here, we generated, analyzed and characterized a high quality circular genome sequence of the *F. tularensis* subsp. *holarctica* strain 12T0050 that caused fatal tularemia in a hare. Besides the genomic structure, we focused on the analysis of oriC, unique to the *Francisella* genus and regulating replication in and outside hosts and the first report on genomic DNA methylation of a *Francisella* strain. The high quality genome was used to establish and evaluate a diagnostic whole genome sequencing pipeline. A genotyping strategy for *F. tularensis* was developed using various bioinformatics tools for genotyping. Additionally, whole genome sequences of *F. tularensis* subsp. *holarctica* isolates isolated in the years 2008–2015 in Germany were generated. A phylogenetic analysis allowed to determine the genetic relatedness of these isolates and confirmed the highly conserved nature of *F. tularensis* subsp. *holarctica.*

## Introduction

*Francisella (F.) tularensis* is a small, highly infectious, Gram-negative, fastidious bacterial pathogen and the causative agent of tularemia (Ellis et al., 2002). Tularemia is a zoonosis that can be transmitted to humans through infected blood-feeding arthropods or by ingestion or inhalation of *F. tularensis*. Human infections with *F. tularensis* occur through skin occulation after handling infected animals and occasional through laboratory infections. *F. tularensis* is listed as a category A bioterrorism agent, because the infectious dose is very low and the resulting febrile disease may be severe to fatal. It requires prompt antibiotic treatment to avoid strong complications (Rotz et al., 2002; Maurin, 2015). The two subspecies *F. tularensis* subsp. *tularensis* and *F.* *tularensis* subsp. *holarctica* are the major causes of tularemia in humans (Kingry and Petersen, 2014). Only the less pathogenic *F. tularensis* subsp. *holarctica* is endemic in Europe (Tärnvik and Berglund, 2003). In Germany and France most human infections are caused by contact with infected European brown hares (*Lepus europaeus*) (Müller et al., 2013; Robert-Koch-Institut, 2015; Moinet et al., 2016). *F. tularensis* subsp. *holarctica* appears to be a re-emerging pathogen in Germany infecting many animal species and arthropod vectors like ticks (Müller et al., 2013; Otto et al., 2015; Robert-Koch-Institut, 2015).

Most *Francisella* species share many biological and genomic attributes, but the genetic and functional differences significantly influence virulence and pathogenicity (Jones et al., 2012, 2014; Ulland et al., 2013). It is known that *F. tularensis* is a facultative intracellular pathogen infecting a wide variety of cells (Ozanic et al., 2015). Following uptake into the macrophage, the bacteria at first reside within a phagosome, but then rapidly escapes into the cytoplasm. Adaptation to the specialized intracellular lifestyle is associated with evolutionary loss of genes for many metabolic pathways, but *F. tularensis* has retained or evolved mechanisms to efficiently acquire essential nutrients within the host (Barker et al., 2009; de Bruin et al., 2011).

All *Francisella* isolates (including *F. tularensis* subsp. *tularensis*, *F. tularensis* subsp. *holarctica*, *F. novicida)* reside in the environment or in a variety of animal hosts. They have small conserved genomes of about 2 Mb. *F. tularensis* strains have a high degree of genetic similarity with an average nucleotide identity of ≥97.7% (Larsson et al., 2009). Nevertheless, differences are apparent between their respective genomes, genome sizes and protein coding genes. Genome sequencing and analysis has been performed on several *F. tularensis* strains, with a limited number of genomes fully assembled and annotated. Although it is known that *F. tularensis* has methylated DNA (Elkins et al., 1999), DNA methylation analysis was not done yet with other previously sequenced strains. The analysis of DNA methylation can elucidate its role in gene regulation. DNA methylation protects the integrity of prokaryote genomes, but also plays a role in chromosome replication, nucleotide segregation, DNA repair, and transcription (Wion and Casadesus, 2006; Murray et al., 2012; Kumar and Rao, 2013). Bacterial DNA from *F. tularensis* (LVS) containing unmethylated CpG motifs triggers an activation of B-cells but no activation when the DNA is methylated (Elkins et al., 1999). The methylation of *F. tularensis* subsp. *holarctica* hence might play a key role in the pathogenic stealth mechanisms of *F. tularensis* subsp. *holarctica* in macrophages (Champion, 2011). Thus, single-molecule real-time (SMRT) sequencing that reveals methylation throughout the genome is a powerful tool for the investigation of this pathogen. We report here the first methylation analysis of a *F. tularensis* genome based on a hybrid assembly using two sequencing technologies (long reads and short reads) and thus high in quality. We assessed the phylogeny of this strain with samples from the same region in North Rhine-Westphalia (Germany) collected in the years 2008–2015. An analysis pipeline was established by using this high quality genome to evaluate the best approaches for short read assembly and genome annotation. The microbial phylogeny of *F. tularensis* subsp. *holarctica* could be generated. In the first step the inclusion into the tree of life was targeted. The classification into the phylogenetic tree of life is necessary to allow for an exact classification of new and unknown bacterial species. In a second step the analysis with MLST^+^ and Parsnp as reference-independent molecular typing tools enabled a novel detailed view on *F. tularensis* subsp. *holarctica* epidemiology for the *F. tularensis* subsp. *holarctica* 12T0050 in the related regional setting.

## Materials and Methods

### Bacterial Strains

The bacterial *F. tularensis* subsp. *holarctica* strain 12T0050 used in the present study as a reference strain was isolated on cysteine heart agar (CHA, Becton Deckinson, BD Heidelberg, Germany) from a carcass of a hare (*Lepus europaeus*) found during an outbreak in 2012 near Herringhausen (North Rhine-Westphalia, Germany). The strain was assigned to clade B.6, subclade B.18 using a set of real-time PCR assays (Robert-Koch-Institut, 2015; Tomaso et al., 2017). Subsequent whole genome sequencing and bioinformatics analysis using CanSNPer^1^, which is an assay for whole genome sequencing based on canonical single nucleotide polymorphisms (canSNPs) based on whole genome sequences developed by Larkeryd et al. (2014) confirmed clade B.6. The cultivation of bacteria from organ specimens was performed on cysteine heart agar at 37°C with 5% CO_2_ for 48 h.

For phylogenetic analysis, bacterial strains were chosen from the collection of strains and sequences maintained at the Friedrich-Loeffler-Institut, Institute of Bacterial Infections and Zoonoses, Jena, Germany. The selection included 14 strains collected in the years 2009–2015 in a region close to the outbreak in 2012 in North Rhine-Westphalia (Germany). All strains were identified by MALDI-TOF MS (Seibold et al., 2007; Müller et al., 2013) and PCR assays and assigned to genetic clades and subclades using real-time PCR assay that target canSNPs and INDELs as described in (Tomaso et al., 2017) and shown in **Table 1**. The whole genome sequences were analyzed with the canSNPer tool (Larkeryd et al., 2014). The reference strain FSC237, *F. tularensis* subsp. *tularensis* SCHU S4 (NC_006570.2), a known human pathogen, was sequenced and assembled to be included as an outlier (**Figure 3**). *F. tularensis* subsp. *tularensis* was cultivated under BSL-3 conditions, *F. tularensis* subsp. *holarctica* strains were handled under BSL-2 conditions in accordance with German biosafety regulations. The isolates were inactivated at 95°C for 20 min.

**Table 1:** *Francisella tularensis* subsp. *holarctica* isolates from North Rhine-Westphalia (Germany) with qPCR and canSNPer results, year of collection, and district of isolation. (x; no result obtained).

Sample ID	Clade, qPCR	Subclade, qPCR	canSNPer	Collection Date	District
09T0179	B.6	B.18	B.51	2009	Geseke
10T0115	B.6	B.18	x	2010	Waltrop
10T0192	B.6	B.18	B.51	2010	Geseke
10T0193	B.6	B.18	B.51	2010	Geseke
11T0309	B.6	B.18	B.49	2011	Soest
12T0002	B.6	B.18	B.45	2012	Huels
**12T0050**	**B.6**	**B.18**	**B.X**	**2012**	**Herringhausen**
12T0062	B.6	B.18	B.62	2012	Lippstadt
15T0012	B.12	B.34	B.26	2015	Hoexter
15T0013	B.6	B.18	B.62	2015	Hoexter
15T0014	B.6	B.18	B.62	2015	Paderborn
15T0016	B.6	B.18	B.11	2015	Lippe
15T0031	B.12	B.34	B.26	2015	Ostwestfalen-Lippe
15T0085	B.6	B.18	B.45	2015	Euskirchen
15T0086	B.6	B.18	B.49	2015	Euskirchen


*Bold lettering is highlighting the isolate 12T0050.*

### DNA Extraction and Genome Sequencing

DNA for whole genome sequencing was prepared from a 10 mL culture in brain heart infusion broth (Brain, Heart Infusion Broth, Sifin, Berlin, Germany), Bacterial cells were harvested after 72 h by centrifugation, and the DNA was purified using QIAGEN Genomic-tip 20/G and a QIAGEN Genomic DNA buffer set kit (Qiagen, Hilden, Germany). DNA quality was examined by using a Qubit 2.0 fluorometer (Life technologies, Germany) and by agarose gel electrophoresis.

### Sequencing, Assembly, Annotation and Genomic Analysis Tools

The isolate 12T0050 was subjected to PacBio sequencing, HiSeq and MiSeq sequencing on Illumina instruments and to Ion Torrent sequencing. IonTorrent Sequencing was performed with standard procedure with the AB library builder on Ion Torrent S5XL with 520 Chip. The genome sequencing analysis of strain 12T0050 was started with SMRT DNA sequencing (McCarthy, 2010) using a PacBio RSII sequencer at GATC Biotech (Germany). Genome assembly was carried out using the HGAP algorithm version 3 (RS_HGAP_Assembly.3) (Chin et al., 2013) implemented in PacBio SMRT portal version 2.3.0. The two SMRT^®^ Cells were pooled together and assembled. Circularization of the genome sequence, represented by a single contig, and merging of the contig was carried out using Circlator (Hunt et al., 2015). The circular contig was polished with the RS_Resequencing.1 protocol available on the SMRT portal v2.3.0. Methylome analysis was done with RS_Modification_detection.1 and Modification_and_Motif_Analysis.1, both are also available in the SMRT portalv2.3.0. After circularization, the sequence was corrected with Illumina MiSeq data for the substitutions due to sequencing errors and for frameshifts to generate “hybrid assemblies” (software used from Boyke Bund, personal communication, available on request) and named 12T0050_FLI.

This optimized sequence of *F. tularensis* subsp. *holarctica* isolate 12T0050_FLI was used to compare the results of various assembly and annotation software solutions. For quality control and trimming of the sequencing reads the programs bbmap/bbduk suite (Bushnell, 2017) and sickle (Schirmer et al., 2015) were used. The following open source assembler programs were tested: SPAdes v. 3.9.1 (Bankevich et al., 2012) (with and without error correction in Bayes Hammer modes supported by GNU-parallelization (Tange, 2011), MaSuRCA 3.1.0 (Zimin et al., 2013), and ABySS 2.0.2 (Simpson et al., 2009). Additionally, the assembler of CLC (CLC Genomics Workbench 9.5.3)^2^ was included, a commercially available software package with quality check and automated preprocessing. Analysis of the generated assembly was performed with QUAST 4.3 (Gurevich et al., 2013) with gene prediction using GeneMarkS Suite and Bandage 0.8.1 (Wick et al., 2015). All three short read sequencing technologies performed equally well, though longer read length in MiSeq data led to a reduction of contig numbers. The assembled contigs were tested with different annotation or gene identification algorithms and compared: Prokka annotation pipeline 1.12-beta in standard settings (Seemann, 2014), Rapid Annotation using Subsystem Technology (RAST) server and Glimmer 3, Glimmer HMM-3.0.3 (Delcher et al., 1999, 2007; Aziz et al., 2008) and AUGUSTUS, an annotation tool, optimized for eukaryotes but also known to produce good annotations with prokaryotes (Delcher et al., 1999, 2007; Stanke et al., 2006; Aziz et al., 2008; Meyer et al., 2008). Samples were tested for contaminations with Kraken version 0.10.6-unreleased (Wood and Salzberg, 2014) and manually curated to exclude samples with high contamination rates. We established a uniform protocol for all data, consisting of SPAdes assembly in the Bayes-Hammer mode, the filtering of contigs (removing contigs smaller than 500 bp and with less than 3 reads coverage) and Kraken testing.

For genomic analysis the origin of replication (oriC) was identified using ori-Finder (Gao and Zhang, 2008). CRISPR loci were searched using the CRISPR Recognition Tool version 1.1 (Bland et al., 2007). Prophage elements were searched by using PHAST (Zhou et al., 2011). Tandem repeats were searched for with a tandem repeat finder version 4.09 (Benson, 1999). Average nucleotide identity (ANI) was calculated with enveomics (Rodriguez-R and Konstantinidis, 2016). CpG islands were detected by EMBOSS in standard settings (Rice et al., 2000). Visualization was carried out using smrtview (Chin et al., 2013), Artemis (Carver et al., 2012), and DNAplotter (Carver et al., 2009). Methylation motif analysis was performed with Rebase (Roberts et al., 2015).

All other strains were subjected only to Illumina HiSeq and/or MiSeq sequencing using the Nextera XT DNA protocol for library preparation (GATC, Konstanz, Germany and BfR, Berlin, Germany). The number of reads after filtering ranged from 0.5 million to 5 million resulting in an average nucleotide coverage of >50 (see **Supplementary Figure S1**).

### Phylogenetic Analyses

To assess the phylogenetic classification of assorted *F. tularensis* subsp. *holarctica* genomes, already published methods based on different bioinformatics approaches using whole genome sequence data were compared. All selected strains were characterized using a combination of independent methods including MALDI-TOF MS, conventional PCR and real-time PCR assays targeting INDEL loci and canSNPs as previously described (Tomaso et al., 2007; Larkeryd et al., 2014). The phylogenetic study included various species and subspecies within the genus, such as *F. tularensis* subsp. *tularensis, F. tularensis* subsp. *novicida, F. philomiragia*, and *F. noatunensis, F. guangzhouensis* (NC_006570.2, NZ_JOOT00000000.1, NZ_CP010427, NC_010336.1, NZ_LTDO00000000.1) retrieved from the NCBI database, as well as strains that are well characterized representatives of different clades of *F. tularensis subsp. holarctica*, i.e., clade B.4 (NC_017463), B.6 (NC_009749) and B.12 (NC_019551). As an outgroup an *Escherichia coli* (NC_002695) and a *Salmonella enterica* genome (NC_003198) were included. All genomes were assessed as assemblies and newly annotated with Prokka to allow for greater comparability.

Taxonomic classification was performed with 16S rRNA gene analysis using MOLE-BLAST (Altschul et al., 1997; Edgar, 2004) with standard settings excluding uncultured samples. The coding sequences for 16S rRNA were extracted with Geneious (Kearse et al., 2012). This was compared to PhyloPhlAn to include the samples into the tree of life. PhyloPhlAn was used with the annotation files resulting from Prokka and performed with standard setting on all samples. These two methods were generated distance matrices on fixed inputs and allowed database independent and sample size independent inclusion in the tree of life. These two methods were compared to SeqSphere and ParsSNP. SeqSphere, a commercially available multilocus sequence typing tool based on the core genome analysis, was used as described (Antwerpen et al., 2015) and compared to the open source software Parsnp v1.2, a command-line-tool for efficient microbial core genome alignment. This program uses SNP detection as part of the Harvest suite in standard setting (Treangen et al., 2014). Core genome of the genome sequences are used to create a multiple meta-alignment file. A maximum likelihood tree was generated with Randomized Accelerated Maximum Likelihood, RaxML (Stamatakis, 2014) in GTRGAMMA model and a bootstrap number of 500. Figures were generated using Dendroscope (Huson et al., 2007). Here we used TempEst for the visualization and analysis of temporally sampled sequence data (Rambaut et al., 2016).

## Results

### Genome Compilation

**FIGURE 1:**
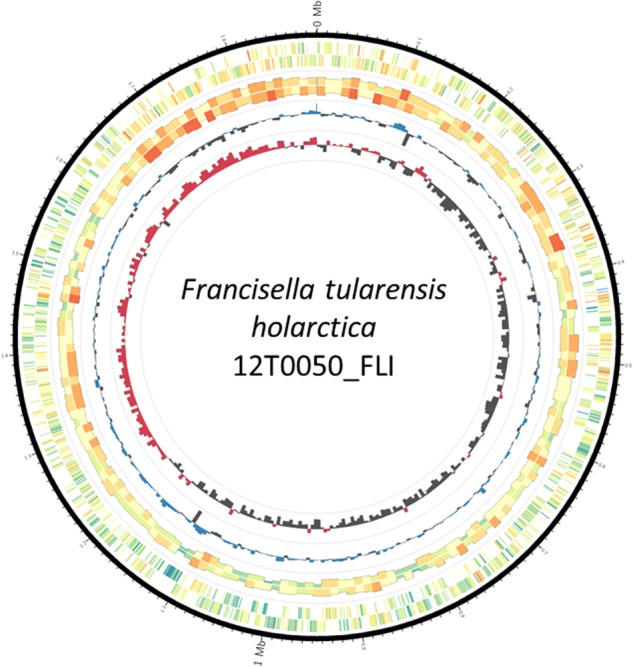
Plot of the complete *Francisella tularensis* subsp. *holarctica* 12T0050. The genome consists of 1890738 base pairs and 2114 predicted coding sequences. The circles represent from the inside: 1, GC skew (red above and black below zero, 10 kb window); 2, GC content (blue above and black below genome average of 32.20%, 10 kb window); 3, strand-specific genome-wide methylation analyzed in 10 kb windows; 4, strand-specific methylation per gene; 5, scale in million base pairs (Mb). Methylation color spectrum goes from blue (minimum, 5%) over yellow (mean, 12.5%) to red (maximum, 23%).

To establish a complete reference genome *F. tularensis* subsp. *holarctica* isolate 12T0050 was sequenced by applying SMRT^®^ Technology. The sequencing approach with 2 flow cells yielded 20 116 reads with a total of 253 596 265 bases, 24 647 reads, an average length of 11477 bases. The total runtime including data transfer and analysis was 172 min. Resequencing resulted in an average reference bases called of 100 and 99.9% concordance and average reference coverage of 114.3. The Hierachical Genome Assembly Process (HGAP) resulted in a single contig of 1890609 bp The GC-content was 32.2%. The chromosome assembly was generated in Circlator and was visualized with the Artemis Comparison Tool (**Figure 1**). High quality genome sequence was made using a hybrid approach involving both long and short reads, whereby the long reads were used for initial assembly and was subsequently corrected using short reads to remove insertion/deletions that occur with long reads alone based assembly approach. The corrections were made accordingly as the Miseq data were mapped with bwa v.0.7.12-r1039, variants were called with varscan v2.3 and the consensus was generated with GATK v.3.7.0-gcfedb67 (Koboldt et al., 2009; Li and Durbin, 2010; Van der Auwera et al., 2013). It is denoted 12T0050_FLI. It is submitted as *Francisella tularensis* subsp. *holarctica* 12T0050_FLI under the BioProject PRJNA422969, BioSample SAMN08201031, Accession number CP025778.

### Genome Assembly

With 12T0050_FLI we assessed the best short-read assembly strategy to set out (see **Supplementary Table S1**). All assemblers were evaluated for contig size, gene content and overall quality. First, the preprocessing was evaluated aiming for quality score based read filtering, base trimming, and removal of contaminations caused by phiX and adapter sequences. Preprocessing (bbduk and sickle) did not result in a significant improvement of the assembly quality in respect to gene content or contig size. The SPAdes assembler in Bayes Hammer mode without preprocessing resulted in a comparable quality to other preprocessing methods. In the assembler comparison the MaSuRCA and ABySS over-and underestimated gene content and genome sizes up to 36%. Robust results were obtained with all sequencer platforms with the SPAdes assembler in Bayes–Hammer mode, which was therefore regarded as the method of choice. An additional quality improvement was generated by excluding contigs that were smaller than 500 bp or had a coverage lower than 3 (using fastgrep.pl)^3^. CLC provided preprocessing and assemblies with comparable good results, but could not easily be included into a workflow with open source programs and was thus excluded.

### Genome Annotation

Genome annotation on the reference genome of 12T0050_FLI with PROKKA resulted in 2114 CDS (Coding DNA sequences, that code for proteins) while RAST and AUGUSTUS resulted in 2141, GlimmerES in 6398 possible open reading frame rather than CDS as shown in **Table 2**.

**Table 2:** Annotated features of the genome *of F. tularensis* subsp. *holarctica* strain 12T0050_FLI, generated with Glimmer, Prokka, RAST and Augustus.

Feature	Glimmer	Prokka	RAST	Augustus
**CDS^∗^**	6398^∗^	**2114**	2141	2141
**rRNA**	30	**10**	48	20
**tRNA**	114	**38**	48	76
**Size**	1890815	**1890815**	1890815	1890815
**GC Content**	32.2%	**32.2%**	32.2%	32.2%


*^∗^Glimmer predicting only possible protein coding regions not annotating afterwards. In bold lettering the recommended software.*

### Genome Analysis: CRISPR, Prophages, cpG Islands and Origin of Replication

In 12T0050_FLI no CRISPR loci were identified with the CRISPR Recognition Tool version 1.1. The gene FTN_0757 of *F. novicida* showed significant sequence similarity to the CRISPR-CAS system protein Cas9 and one homologue was also found in 12T0050_FLI (1 258 283-1 260 798). In these 2515 nucleotides, 586 methylation sites are reported. Additional, 5 of the directly adjacent 5′ positions of this region are methylated. No prophage elements were identified using PHAST. Eighty-seven tandem repeats were identified with the Tandem Repeats finder. Eight unusual cpG island were predicted using EMBOSS and analyzed for methylation (Rice et al., 2000) (**Supplementary Table S2**).

The origin of replication was predicted in a region with 1071 bp located 100 bp proximal in 5′-direction of 4 DnaA box sequences (similar to ttatccaca) with not more than one mismatch to the *Escherichia coli* DNA box. The origin of replication in *F. tularensis* subsp. *holarctica* 12T0050_FLI was set accordingly. Two DNA boxes had the identical sequence tgtggataa and can be presented as a new DnaA box identifier in *F. tularensis* subsp. *holarctica*. It seems to be characteristic for all *Francisella* species according to Blastnt. All features were included in **Figure 1**.

### Base Modification Detection and Methylation Analysis

Methylation is pervasive in *F. tularensis* subsp. *holarctica* isolate 12T0050_FLI. More than 150,000 methylation sites were detected of which 12–40% were methylated. Most sites belong to the Type I Restriction Modification system that recognizes bipartite motifs and cleave at large distances from their binding sites or orphan methylases. Incomplete methylation is typical for most orphan methylases that are suspected to play a major role in regulation of prokaryotic gene expression. Here, the single molecule, real-time sequencing reads were used to map DNA modifications including N6-methyl-adenosine (m6A), N4-methyl-cytosine (m4C) and N5-methyl-cytosine (m5C), see **Table 3**.

**Table 3:** Methylated motifs detected in the strain *F. tularensis* subsp. *holarctica* strain 12T0050_FLI.

Motif	Modified position	Type	% of motif detected	Number of motifs detected	Number of motifs in genome	Mean modification QV	Mean motif coverage
GGTYDKTGV	1	Unknown	38.8%	123	317	41.68	58.33
ADGTACTA	1	m6A	37.01%	104	281	44.12	54.46
GNNNNVNH	1	Unknown	29.61%	97737	330035	42.05	54.80
GBTBNRVGV	1	Unknown	21.12%	814	3855	39.24	56.61
GSVVNNNG	1	Unknown	20.28%	2841	14011	39.48	55.97
GNNNNTBH	1	Unknown	16.28%	20400	125341	39.88	55.61
TNNBASYW	1	Unknown	14.85%	5951	40077	41.31	55.58
VANDYAGYA	2	m6A	13.89%	527	3793	42.41	54.84
CNNNNRNW	1	Unknown	10.95%	24699	225647	40.82	56.15


*A mean modification Quality Value (QV) refers to the level of confidence that a base is methylated. A QV of 30 or higher is considered significant. The mean coverage for all instances where this motif was detected as modified.*

### Comparative Genome Analysis and Visualization

The assembly of 12T0050_FLI represents the first high quality full genome sequence of an isolate with German origin. It shares the common Clade B.6 with the Swedish *F. tularensis* subsp. *holarctica* strain FTNF-002-00, NC_009749. The finishing of the genome of 12T0050_FLI enabled a detailed comparison of genome architecture und gene content. The sequence reads of 12T0050 were mapped to NC_009749 (Kearse et al., 2012). The main part of variable SNPs between NC_009749 and 12T0050_FLI were in non-coding repeat regions. Only two insertions were in coding regions and resulted in reading frame changes: 73 bp in an aspartate alanine antiporter CDS (CYL81_01665) and one 16 bp insertion in an ISO630 family transposase CDS (CYL81_02715).

### Phylogenetic Analysis

**FIGURE 2:**
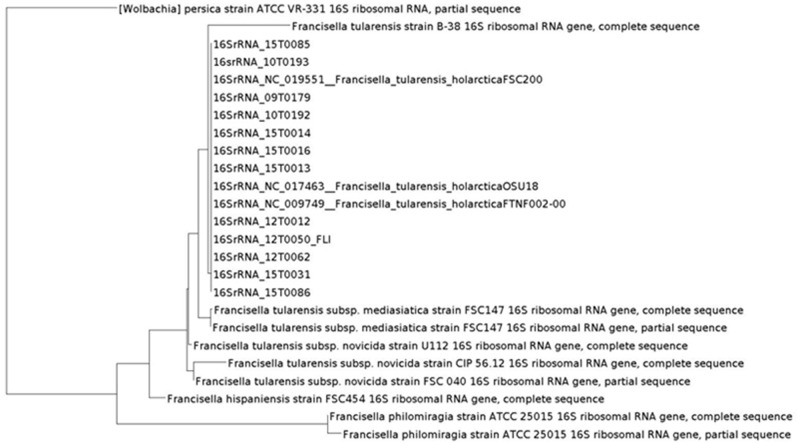
Phylogeny based on 16S rRNA performed with MOLE-BLAST.

**FIGURE 3:**
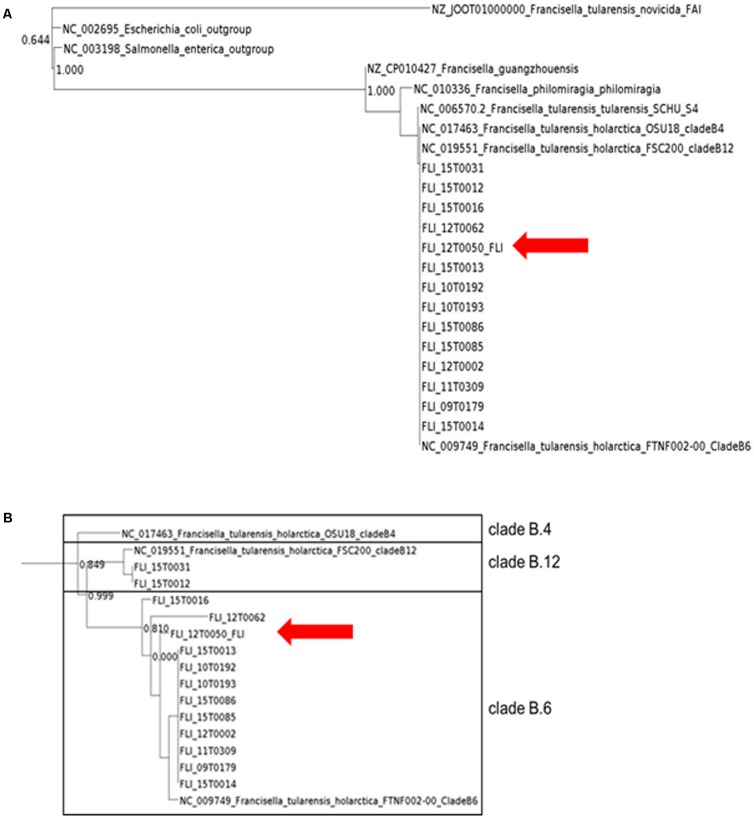
PhyloPhlAn analysis of all *Francisella* isolates using amino acid sequences of more than 400 proteins. The graphic scale equals 2.0 amino acid differences in the overview **(A)** and in the detailed view with *F. tularensis* subsp. *holarctica*, the graphic scale equals 0.001 amino acid difference **(B)**.

In the first step the inclusion into the tree of life was targeted. The classification into the phylogenetic tree of life is necessary to allow for an exact classification of new and unknown bacterial species. The phylogenetic analysis was performed in two steps. First, the taxonomic classification was performed by 16S rRNA analysis with MOLE-BLAST, as the most traditional tool of classification (**Figure 2**). PhyloPhlAn includes a non-redundant database of 400 proteins generated from 3,737 genomes of all microbial taxa to assign microbial phylogeny and putative taxonomy. The software builds phylogenetic trees based on >4,600 aligned amino acid positions, mirroring thus more the changes in the protein sequence and thus functionality than on nucleotide acid changes that might be silent. PhyloPhlAn was able to measure the sequence diversity of all *Francisella* strains allowing even the resolution of the different clades (**Figure 3**). In a second step for closer epidemiological investigations MLST^+^ and Parsnp analysis were compared (**Figure 5**). MLST^+^ was performed with SeqSphere, a multilocus sequence typing tool based on the core genome that was used as described (Antwerpen et al., 2015). Before the usage of Parsnp, a control of the ANI ≥ 97 % is recommended. In the sample group the ANI was between 99.90% (12T0050_FLI/NC_017463) and 99.99% (12T0050_FLI/NC_009749). A deeper analysis was possible with the open source program Parsnp for core genome alignment and SNP detection as part of the Harvest suite in standard settings (Treangen et al., 2014). Common genes distributed across the chromosomes of all genomes analyzed represent the chromosomal core. The MUM segments, representing the core genome to derive SNPs spanned 1.3 Mb. A maximum likelihood tree with the GTRGAMMA model rate of heterogeneity was calculated with RaxML (Stamatakis, 2014) and supported by a bootstrapping test with 500 resamples. Phylogenomic analyses performed in a maximum likelihood (ML) framework using variable coding positions unambiguously identified 137 positions in all genomes for chromosomal sequences. Despite the reduced size of these data sets, unique SNPs were observed for all strains.

Maximum likelihood trees derived from the analysis of chromosomal sequences strongly supported the existence of the three clades B.4, B.6 and B.12. However, the number of SNPs will probably increase with larger numbers of available isolates and sequences of whole genomes. 12T0050 was separated from the reference strain by 17 SNPs to the B.6 strain, 629 to the B.12 strain and 648 to the B.4 strain. A *R*^2^ = 1,89 ^∗^ E-2 value, less than 0.5, suggesting weak clock-like behavior. The regression slope (rate) included negative values.

## Discussion

The sequenced genome 12T0050_FLI enabled a detailed analysis of genome architecture and gene content with bioinformatics tools. Pacific Biosciences DNA sequencing platforms generate long reads that can produce complete genome assemblies, but the sequencing is expensive and error-prone. The Illumina DNA sequencing platform generates accurate but short reads. There is significant interest in combining data from these complementary sequencing technologies to generate more accurate “hybrid” assemblies. Thus, we produced here a hybrid assembly of those two sequencing technologies for an optimal genome with the name 12T0050_FLI. The analysis resulted in a single contig of 1890609 bp and with that a slightly smaller genome than published genome sequences NC_017463 (1895727 bp) or NC_009749 (1890909 bp). The genomic analysis included the comparison to the closest neighbor NC_009749, the identification of the origin of replication, CRISPR regions, prophage regions and the analysis of the methylome. With a two-step phylogenetic analysis we established a new whole genome analysis pipeline that allowed description of a phylogenetic analysis of isolates collected in a circumscribed area in Germany and to elucidate the epidemiological context.

Several genome sequences of *F. tularensis* subsp. *holarctica* are published but only a limited number of genomes has been fully assembled and annotated (Larsson et al., 2005, 2009; Beckstrom-Sternberg et al., 2007; Chaudhuri et al., 2007; Rohmer et al., 2007; Barabote et al., 2009; Champion, 2011; Modise et al., 2012; Sjodin et al., 2012; Svensson et al., 2012; Antwerpen et al., 2013, 2015; Atkins et al., 2015; Busch et al., 2017). The here described assembly is a high quality full genome sequence. It is closely related to the Swedish *F. tularensis* subsp. *holarctica* strain FTNF-002-00, NC_009749 Clade B.6. The genomic sequence 12T0050_FLI was mapped to NC_009749 (Kearse et al., 2012) and 77 SNPs could be called. Most divergent SNPs between NC_009749 and 12T0050_FLI were in non-coding repetitive regions and might be sequencing or assembly errors. Two insertions were in coding regions and resulted in reading frame changes: 73 bp in an aspartate alanine antiporter CDS and a 16 bp insertion in an ISO 630 family transposase CDS both of hitherto unknown effect on the phenotype.

CRISPR elements in bacteria confer protection against bacteriophages; approximately 40% of the bacterial genomes are known to carry CRISPR elements (Barrangou et al., 2007). In 12T0050_FLI no CRISPR loci were *in silico* detected. A CRISPR/Cas9 system has been reported for *F. novicida* (Sampson et al., 2013) and also from other *Francisella* strains, but these systems seem to be non-functional (Schunder et al., 2013). Especially the gene FTN_0757 was found to have sequence similarity to the CRISPR-CAS system protein Cas9 (Sampson et al., 2013). One homologue has also been found in 12T0050_FLI and is extensively methylated (23 %) (CYL81_06580). This coincidence might have major impact on endogenous gene regulation, ultimately promoting both, pathogenesis and commensalism.

Phage therapy is a potential alternative to the use of antibiotics in the up-coming era of drug-resistant pathogens. Especially because of the highly infectious nature of *F. tularensis*, phages might be one of the potential replacement options for antibiotics. Occasionally, phages are also involved in the lateral transfer of mobile DNA elements or bacterial DNA (Canchaya et al., 2003; Golkar et al., 2014). That would be of utmost importance because of the few differences that distinguish the highly virulent form of *F. tularensis* subsp. *tularensis* from *F. tularensis* subsp. *holarctica*. However, no prophage elements were identified using PHAST, indicating that *F. tularensis* subsp. *holarctica* does not host viral infections. That minimizes the alternatives for antibiotics in form of phage therapy, but also the risks of lateral gene transfer. Fortunately, naturally occurring *F. tularensis* strains can be treated with several antibiotics and no tendency to increased resistance has been observed (Tomaso et al., 2017).

The delineated origin of replication, the oriC, appears to be characteristic and unique for *F. tularensis* in general. The origin of replication was predicted in a region of approximately 100 bp upstream in 5′-direction of 4 DnaA box sequences. Three methods, based on DNA asymmetry, the distribution of DnaA boxes and dnaA gene location, were applied to identify the putative replication origins in single replication origin (oriC) in the genome of *F. tularensis* subsp. *holarctica* strain 12T0050_FLI (Mackiewicz et al., 2004). Marker genes commonly observed near the bacterial origin of replication were found near the oriC region (chromosomal replication initiator protein DnaA CDS (CYL81_00005), priA gene (CYL81_009610), recA (CYL81_00060). Two probable DNA-unwinding element (DUE) sites were identified within the shorter oriC region based on its higher A/T composition. Two DNA boxes have the identical sequence TGTGGATAA and can be presented as new DnaA box identifier in *F. tularensis* subsp. *holarctica*. Also both DNA boxes were predicted to be highly methylated at the motif GNNNVNH from hitherto unknown DNA-methyltransferase. This motif is similar to known 6mA methylation motifs such as tttAynnnnngtg from *Clostridium perfringens* or cyayyyyyyctc from *Geopsychrobacter electrophilus*. DNA-methylation without a restriction enzyme is quite common and conserved methylation patterns are evolutionary stable playing an important role in genome replication regulation (Blow et al., 2016). The organisms with the highest local similarity between sequences are *F. tularensis* subsp. *holarctica* and *F. tularensis* subsp. *tularensis* as detected by megablast, (Morgulis et al., 2008). The species with the next most similar sequences are *Gilliamella apicola* and *Vibrio anguillarum*, both belonging to the Gammaproteobacteriacea and are isolated from in bee and fish with coverage of only 8–17% of the regions and approximately 80% identity. This analysis provides insight into the high conservation of this region. This oriC is unique for the whole *F. tularensis* group. Also two probable DNA unwinding regions that are A/T rich could be identified in the oriC region (see also GC-skew in **Figure 1**). The enrichment of methylated GATC motifs in the origin of replication indicates that DNA methylation may regulate genome replication in a manner similar to that seen in *Escherichia coli*. Interestingly, only the methylation motif VANDYAGYA could be also identified in the strain *Enterococcus faecium* isolate 2014-VREF-63 (Rebase (Roberts et al., 2015)).

In highly clonal species that share the bulk of their genomes (>95%), such as *Francisella*, subtle changes, especially those that may alter gene expression such as for example methylation, are likely to have a significant effect on the pathogen’s biology (Champion, 2011). The methylation of *F. tularensis* subsp. *holarctica* might also play a key role in the pathogenic stealth mechanisms of *F. tularensis* subsp. *holarctica* in macrophages. We found that methylation is pervasive in *F. tularensis* subsp. *holarctica* strain 12T0050_FLI as in most bacterial species. In total, 9 methylated motifs were identified being in the normal range of motifs compared to 0–19 methylated motifs per organism in 230 other prokaryotes (Blow et al., 2016). Of these motifs 10–39% where methylated. The predominant base modification type detected was m6A in 2 cases; all others could not be identified. The identification of m6A methylated motifs is consistent with the high abundance of this modification type in the databases. They are also of special interest because m6A are known epigenetic signals for DNA-protein interactions (Wion and Casadesus, 2006). The other modifications, especially m5C, might be underestimated due to the lower sensitivity of SMRT sequencing to these modifications. At least two methyltransferases (MTases) are predicted to be able to perform m6A methylation. The predicted methylation sites are higher methylated, but in most of them no known proteins were annotated. Most methylation sites belong to the Type I restriction modification system that recognizes bipartite motifs and cleave at large distances from their binding sites or orphan methylases. Incomplete methylation is typical for most orphan methylases that are suspected to play a major role in regulation of prokaryotic gene expression. This might indicate a methylation based prokaryotic gene expression in *F. tularensis* subsp. *holarctica* being evolutionary older than the assumed defense function of the restriction modification systems and which could be a reason for the genomic structure of *F. tularensis* subsp. *holarctica* (Blow et al., 2016). In *Escherichia coli* the chromosome replication and nucleotide degradation is dependent on the methylation status of 11 GATC sites near the origin of replication (*oriC*), whereas hemimethylated origin sites are inactive. In *Salmonella*, a close relative of *F. tularensis* subsp. *holarctica* and belonging also to the Gammaproteobacteriacea, the key control in pathogenic virulence is the regulation of virulence genes by methylation of a DAM methylase.

CpG island prediction was performed with standard settings of EMBOSS (Rice et al., 2000), resulting in 8 cpG islands of unusual CG composition. Additional to the methylation pattern the cpG islands were analyzed. Eukaryotic DNA methylation is known to be specific for cytosines in cpG sequences. However, the protective function of DNA methylation is similar in eukaryotes and prokaryotes and it is proposed that cpG islands are associated with promoters that influence DNA replication and other functionalities (Antequera and Bird, 1999). Depending on the repetitive sequence, methylation can significantly enhance or reduce its genetic stability (Nichol and Pearson, 2002). Bacterial DNA from *F. tularensis* (LVS) containing unmethylated CpG Motifs triggers an activation of B-cells but no activation was triggered, when the DNA was methylated (Elkins et al., 1999). Eight unusual cpG island were predicted using EMBOSS (Rice et al., 2000) (see **Supplementary Table S2**). The high methylation hints at a regulation which has to be explored by functional analysis. This regulative mechanism might help to evaluate the pathogenic pathways of *F. tularensis* subsp. *holarctica* strain 12T0050 (Elkins et al., 1999).

To assess the phylogenetic origin of *F. tularensis* subsp. *holarctica* strain 12T0050 we investigated 14 strains of the collection at the Friedrich-Loeffler-Institut. They were collected in the years 2009–2015 in North Rhine-Westphalia, Germany, in the same region as isolate 12T0050 and were found up to 200 km apart. Whole genome sequencing with short sequences was performed. *Francisella* has a very difficult to assemble genome containing a repeat size of greater than 7 kbp (Class III) (Koren et al., 2013). Especially, the *Francisella* Pathogenicity Island that is reported to be duplicated in all the subspecies of *F. tularensis* (*F. tularensis* subsp. *holarctica*, *tularensis*, and *mediasiatica*) but is present using a single copy in *F. novicida* and *F. philomiragia* (Broms et al., 2010), is assembled into one contig using short sequence assemblers. With 12T0050_FLI, we were able to determine the currently best short-read assembly and annotation software. Although quality trimming and preprocessing with sickle was reported to reduce the transposon related artifacts as in Nextera XT kits from Illumina, in the preprocessing these seem to have only minor effects on the data set used here. Also bbduk, which is a tool for quality trimming and preprocessing, showed only minor effect. SPAdes in the Bayes Hammer mode without any further preprocessing was the method of choice for sequencer platforms. It is known that raw sequencing files contain contaminations (Mukherjee et al., 2015). A quality improvement was generated by excluding contigs that were smaller than 500 bp or with coverage lower than 3. Kraken analysis that was included into the workflow as a control for contamination and the analysis of the excluded contigs showed indeed a high percentage of contaminated sequences. The classification into the tree of life allows newly sequenced and unknown species to be classified correctly as was shown recently for new *Francisella* species (Rydzewski et al., 2014). Four protein annotation software were compared for analyzing the coding DNA sequences (CDS). The Prokka pipeline provided fast, easy and robust handling and could be easily curated with Artemis (Carver et al., 2012). The annotated content of 10 rRNA is the same as the previously published data of the complete genomes NC_007880 (LVS) and NC_008369 (OSU18), and is more reliable than the often misassembled rRNA annotations in short read assemblies. Thus, after SPAdes assembly, contig filtering and Kraken analysis, the Prokka annotation could easily be included. This pipeline was used as a uniform protocol for the here treated data. This was followed by phylogenetic analysis, starting with MOLE-BLAST and PhyloPhlAn to allow a classification into the phylogenetic tree of life and followed by closer epidemiological investigations using MLST^+^ and Parsnp analysis.

To benchmark our whole genome comparisons with the prior literature, a phylogeny based on 16S rRNA gene sequences, historically the most frequently applied phylogenetic marker was generated with MOLE-BLAST (Altschul et al., 1997) that relies on the curated, updated and comprehensive database of NCBI. Thus, generated phylogenies allow newly sequenced organisms to be included in the tree of life using a broad data basis, as could be shown here in a tree including our dataset (**Figure 3**). The coding sequences for the 16S rRNA could be extracted with geneious (Kearse et al., 2012) or MOLE-BLAST, which could be used with the assembled files. This consistently revealed only few differences between the *F. tularensis* subsp. *holarctica* strains and clustered together all of our strains into the *Francisella* group. The 16S rRNA phylogeny allowed us a simple and fast classification to the genus, species, and subspecies level (data in **Table 1** and Tomaso et al., 2017).

**FIGURE 4:**
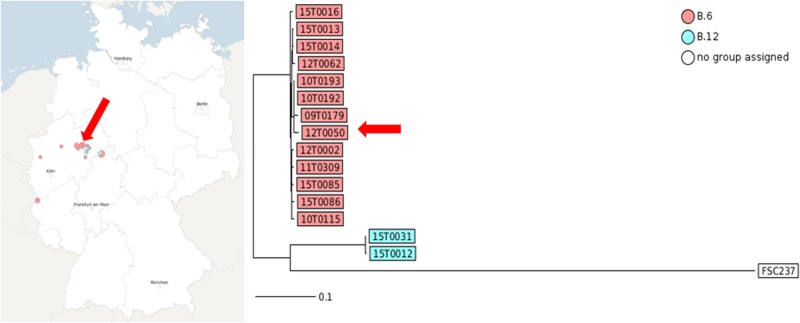
MLST^+^ of the strains showing the distance based on 1147 core genome MLST loci generated with SeqSphere. The graphic scale equals 0.1 difference of a MLST locus. Red colored isolate numbers indicate assignment to clade B.6, blue to clade B.12. The arrow marks the position of 12T0050.

For more profound characterization, we utilized PhyloPhlAn, which is a software tool for accurately determining taxonomic identities and evolutionary relationships of novel microbial genomes (Segata et al., 2013). Results from PhyloPhlAn are based on substantially more data compared to 16S rRNA or even the MLST^+^ approach presented in **Figure 4**. The program determines the protein sequence diversity and improves consistency between phylogenetic and taxonomic groupings. PhyloPhlAn achieves taxonomic levels of high precision from phyla to species level but also reflects a substantial number of provisional clades, as could be shown also here in the genus of *Francisella*. PhyloPhlAn was able to define distinct genotypes for *F. tularensis* subsp. *holarctica* for representatives of all major subclades, B.4, B.6, and B.12, respectively. The results of MOLE-BLAST and PhyloPhlAn are in good concordance with the data from MALDI-TOF and PCR and with all the results of conventional typing methods.

**FIGURE 5:**
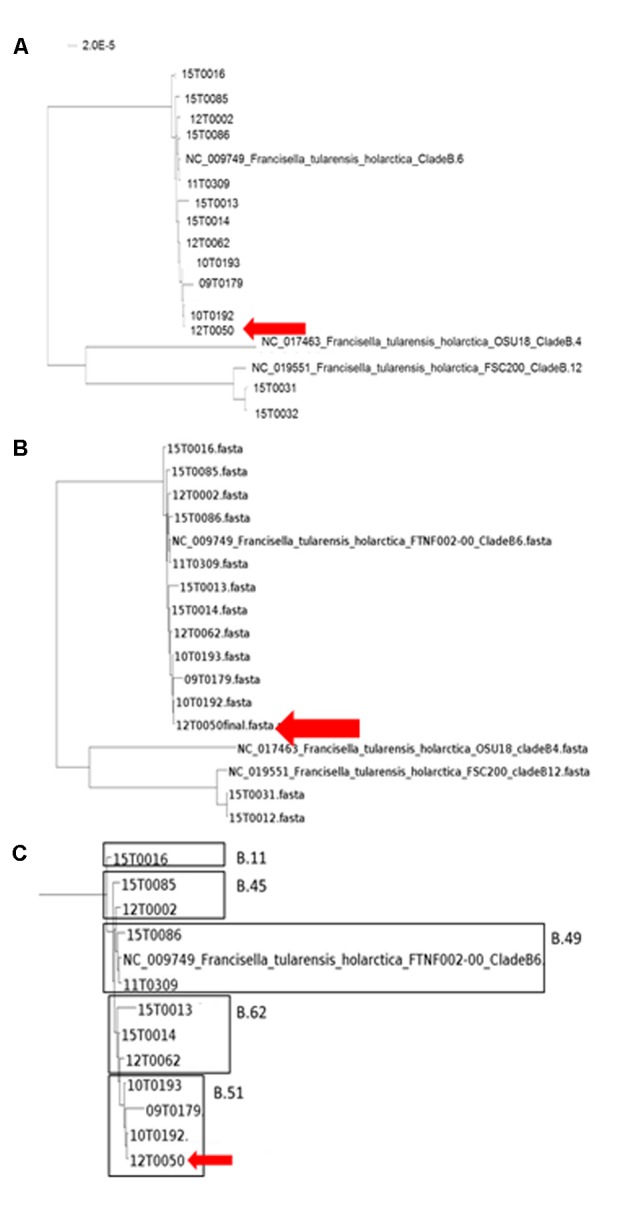
Neighbor-Joining Phylogenetic Tree of the ParSNP core genome SNP analysis **(A)**. The trees were statistically evaluated with RaXML with a bootstrap analysis with 500 bootstraps. (**B** and detailed view **C**, compared to canSNPer analysis)

To perform a more detailed epidemiological analysis the core genome based MLST^+^ method of SeqSphere (Antwerpen et al., 2015) and the core-genome multi-aligner Parsnp of the Harvest suite were compared (Treangen et al., 2014). Canonical single nucleotide polymorphisms (canSNPs) assays (qPCR and canSNPer) and MLST^+^ distinct clusters of genotypes allowed to identify phylogeographic patterns of *F. tularensis* (Pilo et al., 2009; Svensson et al., 2009; Vogler et al., 2009, 2011; Chanturia et al., 2011; Gyuranecz et al., 2012; Karlsson et al., 2013; Antwerpen et al., 2015).

The MLST^+^ tree analysis is based on whole genome sequencing, core genome SNP calling, and defined 1147 MLST loci. The application of MLST^+^ revealed two different genotypes with different degrees of relatedness among the investigated isolates. This was in line with laboratory qPCR data (see **Table 1**). Isolate 15T0016 was forming an outgroup. The assignment to genetic clades was nearly identical to results obtained with real-time PCR assays targeting canSNPs and INDELs, but no spatial and temporal clusters could be shown.

Finally, Parsnp analysis was performed. When using genomic variants for phylogenetic analysis, comparative genomics, or outbreak investigations, it is critical to properly evaluate the variant calling method and also to re-evaluate them on a regular basis (Olson et al., 2015). With a growing number of genome sequences the multiple alignments of homologous sequences followed by inference of a tree scale poorly. Therefore, independent ‘alignment-free’ methods should be preferably used (Chan and Ragan, 2013). Parsnp combines the advantages of both, whole-genome alignment and read mapping. Parsnp scales to thousands of closely related genomes. To achieve this scalability, Parsnp is based on a suffix graph data structure for the rapid identification of maximal unique matches (MUMs), which serve as a common foundation to many pairwise and multiple genome alignment tools (Treangen et al., 2014). Following the Harvest suite for rapid core-genome alignment was used. For alignment a maximum likelihood tree with the GTRGAMMA model rate of heterogeneity was calculated with RaxML (Stamatakis, 2014) and supported by a bootstrapping test with 500 resamples. This method provided the highest resolution and was mostly congruent with the qPCR and canSNPer result. The groups B.11, B.45, B.49, B.51, and B.64 were identified.

Here, only random temporal and spatial distribution patterns can be described due to the small sample number. But we could achieve a more detailed differentiation for example for the three strains 12T0062, 15T0013 and 15T0014. The fact that the strain 12T0062 clustered together with 15T003 und 15T0014 was interesting because the canSNPer clustered them together. This phylogenetic clustering will be more precise because more data points are included. The placement of isolate 12T0062 was allocated on a separate branch in the MLST^+^ and the PhyloPhlAn trees, and had an older common ancestor in the Parsnp/RaxML tree, whereas it was assigned to a branch by the typing methods based on the canSNPer approach. Also the closer resolution of the strains 10T0193, 09T0179, 12T0050 and 10T0192 that cluster in the canSNPer and qPCR analysis in one cluster form in this analysis a much more diverse group.

There is a need for an automated approach based on whole genome sequencing data. The here described pipeline is independent of a reference genome and allows analysis on varying sampling sizes, thus meeting the need of phylogenomics. A bioinformatics pipeline consisting of SPAdes, Prokka, PhyloPhlAn and Parsnp was here evaluated. While the study contains relatively few strains taken from a small area it bears the powerful option to directly broaden the impact by inclusion of data from other countries. It also opens up the option to consider host specific differences. This higher resolution can result in a more detailed view of epidemiology, including the phylogenetic aspect. The broader data basis for our placements compared to the canSNPer or MLST^+^ models allows reclassifying diverged clades by high-resolution protein sequence analysis and more nucleotide sequence variations. An advantage of the here described pipeline approach is congruent with the qPCR approach but leads to a higher resolution. Thus, for future rapid routine whole genome sequencing can be used. When used with more isolates it might be even possible to establish a higher spatial and temporal resolution and thus to generate a highly standardized nomenclature for subpopulations. Besides, with relatively minor genetic differences found in the genomic analysis of *F. tularensis* subsp. *holarctica* isolate 12T0050 is highly conserved among the collected strains. *F. tularensis* subsp. *holarctica* strains investigated here showed only very low genetic evolution in the observation period. This is especially evident with a sequence comparison to NC_009749 that had been isolated more than 20 years ago (1997) and is geographical more than 1000 km away. To assess the evolution of *F. tularensis* subsp. *holarctica* we calculated the rates of molecular evolution that are the product of the number of mutations that arise per replication event, the frequency of replication events per unit time and the probability of mutational fixation (Duchene et al., 2016). The regression slope (rate) found here included negative values, suggesting that these rates are either too low or not enough with the here used data set to allow reliable rate estimation (data not shown). Similar data are obtained from *Mycobacterium leprae* relying on a set displaying moderate to strong temporal signal (Duchene et al., 2016). This indicates that *F. tularensis* subsp. *holarctica* is a highly specialized and successful pathogen whose evolution might have reached a dead end such as in *Clostridium chauvoei* (Rychener et al., 2017). Genomics of *F. tularensis* subsp. *holarctica* strain 12T0050 and comparative genomics with other *Francisella* show a remarkable similarity in gene content despite the ecological and phenotypic diversity.

## Conclusion

Here we introduce a high quality sequence of a *F. tularensis* subsp. *holarctica* strain. This genome represents a unique oriC sequence, interesting Cas9 regions, and prophage regions and so far unknown methylation pattern. Additionally, we evaluated and established an analysis pipeline for *F. tularensis* subsp. *holarctica* in Germany. We performed phylogenetic analysis on different levels, thereby linking existing 16S rRNA data with PhyloPhlAn and core genome SNP analysis with MLST^+^ and Parsnp. These methods to assign microbial phylogeny and putative taxonomy using proteins (PhyloPhlAn) and SNPs (Parsnp) proved to be versatile in the epidemiological assessment of *F. tularensis* subsp. *holarctica* in North Rhine-Westphalia, Germany. A diagnostic whole genome sequencing pipeline was established and evaluated. This novel analysis allows a detailed classification, a very precise placement and the utilization of readily available whole genome data, independent of databases and reference genomes.

## Author Contributions

HT has conceived the study, provided strains, strain information, and metadata to the samples. AB performed bioinformatics analysis of genomes, assembly and phylogenetic relationship. PT took part in the phylogenetic relationship analysis. PT and EZ provided bioinformatics, informatics support and data management. MP provided isolates and geographic information. HB, KN, and JG provided knowledgeable discussion and inclusion on bioinformatics pipelines within the framework of the project Ess-B.A.R. HT, SAD, HH, and HN took part in the study design and project discussion.

## Conflict of Interest Statement

The authors declare that the research was conducted in the absence of any commercial or financial relationships that could be construed as a potential conflict of interest.

## Footnotes

1 https://github.com/adrlar/CanSNPer

2 https://www.qiagenbioinformatics.com/

3 http://nebc.nerc.ac.uk

## Supplementary Material

**FIGURE S1 |** Bioinformatics pipeline.

**TABLE S1 |** Comparison of assembler preprocessing with bbduk and sickle and assembler comparison of the 12T0050 assembly with SPAdes, AByss, MaSuRCA and CLC.

**TABLE S2 |** Predicted CpG island with number of methylated sides and percentage CpG island prediction with standard settings of EMBOSS, with an average methylation percentage between 12–40% the predicted methylation sides are significantly methylated, but for most of them no known proteins were annotated.
