# Supplementary figures and images for: Revisiting Francisella tularensis subsp. holarctica, Causative Agent of Tularemia in Germany With Bioinformatics: New Insights in Genome Structure, DNA Methylation and Comparative Phylogenetic Analysis
Source: Front Microbiol. 2018 Mar 13;9:344. doi: 10.3389/fmicb.2018.00344 (PMC5859110; doi:10.3389/fmicb.2018.00344)

## Slide 1
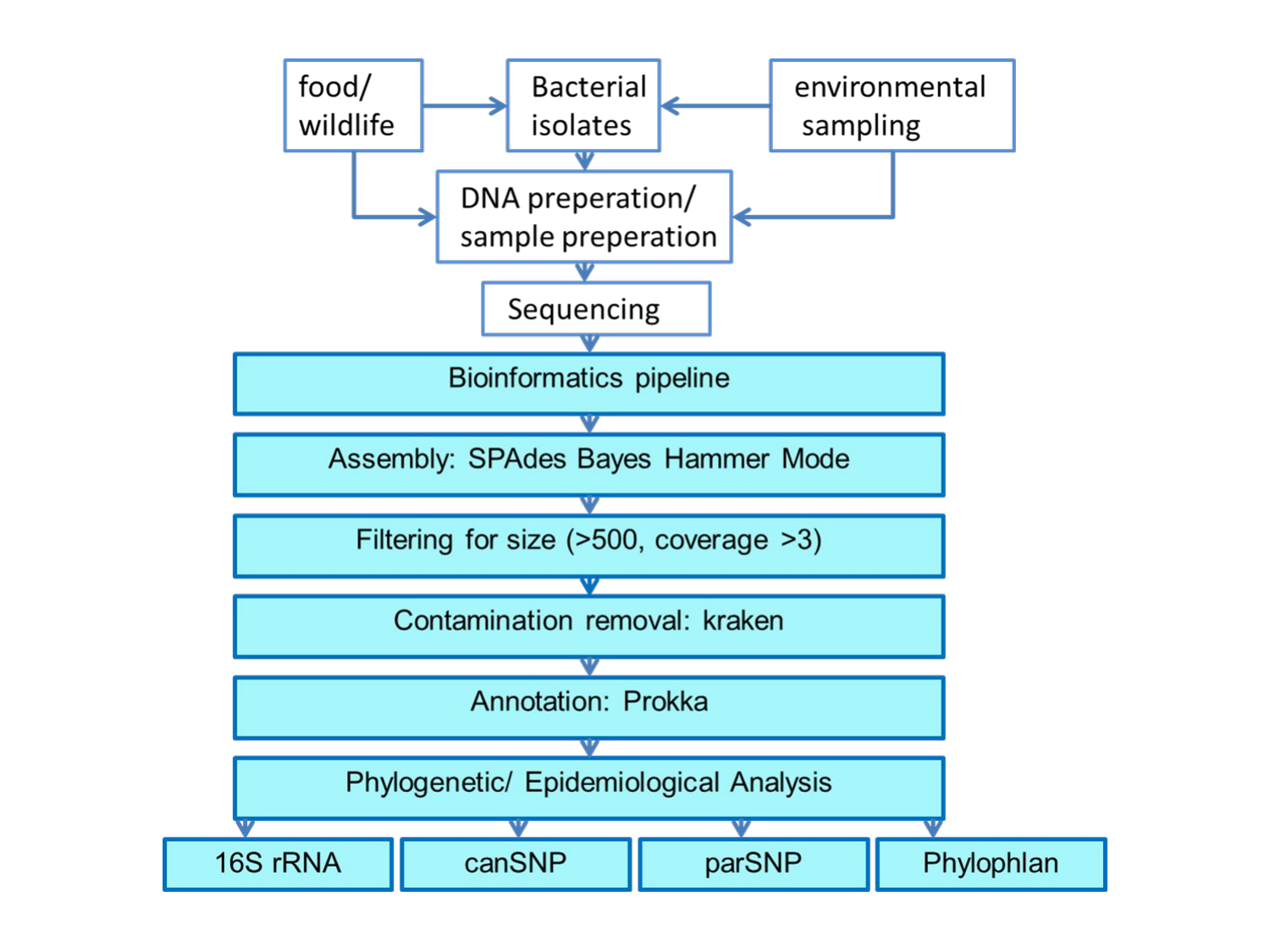

Supplement: Supplementary file 1 [file Presentation_1.pptx]
