# Supplementary material for: Revisiting Francisella tularensis subsp. holarctica, Causative Agent of Tularemia in Germany With Bioinformatics: New Insights in Genome Structure, DNA Methylation and Comparative Phylogenetic Analysis
Source: Front Microbiol. 2018 Mar 13;9:344. doi: 10.3389/fmicb.2018.00344 (PMC5859110; doi:10.3389/fmicb.2018.00344)
Supplement: Supplementary file 2 [file Table_1.docx]

**Table 1 legend:** *F. tularensis*subsp*. holarctica* isolates from North Rhine-Westphalia (Germany) with qPCR and canSNPer results, year of collection, and district of isolation. (x; no result obtained)

**Table 1**

| **Sample ID** | **Clade, qPCR** | **Subclade, qPCR** | **canSNPer** | **Collection Date** | **District** |
| --- | --- | --- | --- | --- | --- |
| 09T0179 | B.6 | B.18 | B.51 | 2009 | Geseke |
| 10T0115 | B.6 | B.18 | x | 2010 | Waltrop |
| 10T0192 | B.6 | B.18 | B.51 | 2010 | Geseke |
| 10T0193 | B.6 | B.18 | B.51 | 2010 | Geseke |
| 11T0309 | B.6 | B.18 | B.49 | 2011 | Soest |
| 12T0002 | B.6 | B.18 | B.45 | 2012 | Huels |
| **12T0050** | **B.6** | **B.18** | **B.X** | **2012** | **Herringhausen** |
| 12T0062 | B.6 | B.18 | B.62 | 2012 | Lippstadt |
| 15T0012 | B.12 | B.34 | B.26 | 2015 | Hoexter |
| 15T0013 | B.6 | B.18 | B.62 | 2015 | Hoexter |
| 15T0014 | B.6 | B.18 | B.62 | 2015 | Paderborn |
| 15T0016 | B.6 | B.18 | B.11 | 2015 | Lippe |
| 15T0031 | B.12 | B.34 | B.26 | 2015 | Ostwestfalen- Lippe |
| 15T0085 | B.6 | B.18 | B.45 | 2015 | Euskirchen |
| 15T0086 | B.6 | B.18 | B.49 | 2015 | Euskirchen |
