# Supplementary material for: Revisiting Francisella tularensis subsp. holarctica, Causative Agent of Tularemia in Germany With Bioinformatics: New Insights in Genome Structure, DNA Methylation and Comparative Phylogenetic Analysis
Source: Front Microbiol. 2018 Mar 13;9:344. doi: 10.3389/fmicb.2018.00344 (PMC5859110; doi:10.3389/fmicb.2018.00344)
Supplement: Supplementary file 3 [file Table_2.docx]

**Supplementary data**

**Supplementary Table 2 legend:** Predicted CpG island with number of methylated sides and percentage CpG island prediction with standard settings of EMBOSS, with an average methylation percentage between 12-40 % the predicted methylation sides are significantly methylated, but for most of them no known proteins were annotated.

**Supplementary Table 2:**

| **Length** | **start** | **stop** | **Methylated sides** | **Percentage of methylated sides** |
| --- | --- | --- | --- | --- |
| 204 | 119639 | 119842 | 77 | 38 % |
| 881 | 120236 | 121116 | 401 | 46 % |
| 291 | 1123196 | 1123486 | 116 | 40 % |
| 535 | 1123654 | 1124188 | 187 | 35 % |
| 246 | 1229693 | 1229938 | 81 | 33 % |
| 294 | 1347177 | 1347470 | 129 | 44 % |
| 246 | 193352 | 193597 | 91 | 37 % |
| 294 | 1210836 | 1211129 | 138 | 47 % |
